# Psychometric Properties of the Spanish Version of the Highly Sensitive Child Scale: The Parent Version

**DOI:** 10.3390/ijerph19053101

**Published:** 2022-03-06

**Authors:** Borja Costa-López, Nicolás Ruiz-Robledillo, Natalia Albaladejo-Blázquez, Monika Baryła-Matejczuk, Rosario Ferrer-Cascales

**Affiliations:** 1 Department of Health Psychology, University of Alicante, 03690 Alicante, Spain; borja.costa@ua.es (B.C.-L.); natalia.albaladejo@ua.es (N.A.-B.); 2 Institute of Psychology and Human Sciences, University of Economics and Innovation, 20-209 Lublin, Poland; monika.baryla@wsei.lublin.pl

**Keywords:** sensory processing sensitivity, environmental sensitivity, highly sensitive child scale, psychometric properties, validity, reliability

## Abstract

Environmental sensitivity is the ability to perceive, register and process information about the environment, which differs among children and adolescents. The Highly Sensitive Child (HSC) scale has been used to assess environmental sensitivity in youngsters. The HSC scale is a short and 12-item adapted version of the Highly Sensitive Person (HSP) scale. The aim of this pilot study is to transculturally adapt the Highly Sensitive Child (HSC) scale, and to analyze its factorial structure, reliability and validity. First, a transcultural adaptation was conducted by bilingual experts. Second, once the questionnaire was translated, the psychometric properties were analyzed. The Spanish version of the HSC scale was administered to parents answering about information of 141 children between 6 and 10 years old. The Spanish version of the Emotionality, Activity and Sociability Survey (EAS) was also applied. The results of the confirmatory factor analysis confirmed the three-factor structure of sensitivity in our Spanish sample. This structure included the following dimensions: (1) Ease of Excitation (EOE), (2) Low Sensory Threshold (LST), and (3) Aesthetic Sensitivity (AES). Moreover, both Cronbach’s *α* and McDonald’s *ω* values indicated that the Spanish version of the HSC scale was a reliable measure of environmental sensitivity, as a general factor of sensitivity (*α* = 0.84), and even in its three dimensions: EOE (*α* = 0.86), LST (*α* = 0.77) and AES (*α* = 0.73). Finally, the correlations for convergent validity showed positive associations, especially among the three dimensions of SPS and Emotionality (EOE r = 0.351; LST r = 0.274; AES r = 0.259), which was one of the domains of the EAS survey. The pilot study provided interesting results, which showed a reliable and valid replication of the original structure of sensitivity in the Spanish samples.

## 1. Introduction

According to the environmental sensitivity’s meta-framework, humans have a survival function of processing information, which allows an adaptation to contexts [1,2]. This makes individuals differ in their sensitivity to environmental influences with some being more or less affected by negative and positive exposures [2]. Regarding the scientific literature, it has provided research related to the variability in environmental sensitivity [2]. In fact, according to researchers’ proposals, three main theories have been developed to describe what entails sensitivity in humans [3,4,5]. The first one is the Sensory Processing Sensitivity (SPS) theory, which is based on a personality perspective [6]. SPS has been presented as a manifestation of environmental sensitivity, and people characterized by a high level of this trait are referred to as highly sensitive [2,6,7]. Moreover, Aron [8] distinguishes four aspects of high sensitivity: (a) great awareness of subtleties, (b) overstimulation, (c) depth of information processing, and (d) high emotional reactivity [9,10]. Furthermore, as claimed by Aron [3], it is estimated that about 30% of the society can present a high manifestation of SPS [11]. This theory also suggests that SPS is a relatively stable personality trait that is shaped from childhood to adulthood while individuals interact with the environment [2,3].

Additionally, in relation to a genetic framework, Belsky [4] has proposed the Differential Susceptibility Theory (DST), which highlights that environmental sensitivity has been defined as a continuum that represents an individual’s sensitivity not only to both negative and positive external environment influences, but also to internal stimuli, for better and for worse, in relation to the perception to respond to situational demands [2,6,7,12,13]. In this regard, high SPS could be manifested as a greater reactivity to environmental stimuli [1,2,14]. Additionally, on the other hand, as stated by Boyce and Ellis [5] in their Biological Sensitivity to Context (BSC) theory, humans present a neurobiological predisposition, suggesting that the environment affects individuals who are physiologically highly reactive [2]. Thus, given the relevant contribution of these three frameworks, researchers agreed that sensitive individuals differ in their responses to both adverse and supportive aspects of the environment [2].

As recent research has further concluded, the construct of environmental sensitivity appears to be formed by a variety of dimensions, instead of referring to it simply as the sensitivity toward sensory stimuli, considering a one-factor structure [7]. Some studies, which have applied scales for assessing environmental sensitivity, have pointed out the presence of three factors [1,15]: (1) the Low Sensory Threshold (LST), which reflects an unpleasant sensory arousal to external and internal stimuli; (2) Ease of Excitation (EOE), which indicates that people with high sensitivity could be easily overwhelmed by external and internal demands; and (3) Aesthetic Sensitivity (AES), which refers to the aesthetic awareness, by noticing subtleties in the environment. Other authors demonstrated the multidimensionality that environmental sensitivity likely shows, establishing different factor structures composed of two, three and four dimensions in psychometric studies in Europe, Asia and Central America [16]. Nevertheless, to date, only Weyn et al. [17] have reported cross-cultural comparisons of environmental sensitivity between the U.K. and Belgium young children.

Regarding the measurement of environmental sensitivity across genders and ages, previous results indicated minor differences between females and males, presenting girls and early adolescents with a significantly higher level of sensitivity than boys and late adolescents [17,18,19]. However, these studies were only conducted with children and adolescents from the U.K., so more research is needed to ensure that these results are interpretated in the same way [1,17].

Thus, the Highly Sensitive Child Scale (HSCS) was developed based on the assumptions of the meta-framework for the concept of environmental sensitivity, especially its creation from the personality perspective of the SPS theory [2]. The HSCS includes 12 items in which important aspects of environmental sensitivity are captured [1]. In fact, a parent-report format in the HSCS is available to assess environmental sensitivity of children in kindergarten and primary education. For this purpose, items were altered and questions rephrased in such a way that parents referred to the behavior they observed in their child. In this sense, Pluess et al. [1] investigated the factorial structure of the original HSC scale, reflecting a bifactorial model. This structure suggests that both the total sensitivity score and the three specific dimensions are important to measure environmental sensitivity [1]. Moreover, recent studies show that the HSC scale presents adequate psychometric properties, considering reliability, convergent validity, and factor analysis in several methodological studies [1,17,20].

In addition, translations of the HSP and HSC scales were carried out using a large number of languages [7]; Dutch, Italian, German, Turkish, Japanese and Icelandic versions are available with adequate psychometric properties [17,21,22,23,24]. In Spain, Chacón et al. [16] validated the first instrument to assess environment sensitivity in adults. In this study, the structure of the questionnaire resulted in no changes in the items, adopting the highest level of linguistic, cultural and conceptual equivalence with the original questionnaire [16]. However, the factor structure and psychometric properties of the HSC scale have not yet been tested in Spanish children.

To date, most researchers agree that environmental sensitivity presents difficulties in its identification [25]. Some research studies reported many sensitivity factors as markers of environmental sensitivity (such as cortisol reactivity, negative emotionality or the 5-HTTLPR gene) [26,27,28]. In fact, it is suggested that the HSC scale could be a more reliable marker of environmental sensitivity than other traditional susceptibility factors, such as temperament [17,20]. However, to date, it is difficult to describe the hypothesis in which the phenotypic trait of environmental sensitivity is directly represented [1]. Therefore, brief and easily applied assessment tools in various contexts can facilitate early detection and intensity in several areas (e.g., the school environment, mental health centers, or family contexts).

Several studies found that HSC scale plays a role of prediction in treatment response to a universal school-based resilience promoting intervention, with children reaching benefits when scoring highly in the HSC [17]. Health and quality of life implications of sensory processing sensitivity on children’s lives highlight the need to promote early identification, since it could surely refer to effects in the evolutive development of youngsters. Brief and easily applied assessment tools in several contexts can ease the detection of high levels of SPS due to the lack of instruments to evaluate this phenotypic trait of environmental sensitivity. Moreover, the applicability of this instrument may provide us with essential information about how environmental sensitivity is manifested in our cultural context.

The present pilot study aims to conduct a transcultural adaptation of the Spanish version of the Highly Sensitive Child Scale and to analyze the psychometric properties of this instrument in Spanish children. To attain this goal, the HSC scale factor structure, internal consistency, and convergent validity of the Spanish version are examined.

## 2. Materials and Methods

### 2.1. Design

We carried out an instrument cross-cultural validation pilot study. We adapted the HSC scale to Spanish in seven stages, according to the Protocol for the Translation of Questionnaires [29]. Firstly, we conducted a linguistic process in the Spanish context for the first five stages. Consequently, the two final stages were dedicated to analyzing the psychometric properties and examined the structure between the Spanish version and the original instrument [29].

### 2.2. Linguistic Validation

The process of the forward- and back-translation and the linguistic adaptation for the HSC scale was carried out in five stages, as indicated in the protocol [29,30,31]. First, we conducted a direct conceptual translation of the original English language version of the HSC scale into Spanish using two bilingual translators, according to the protocol provided by Michael Pluess (the original author). These translators were fluent in both English and Spanish, Spanish being their mother tongue and not linked to the project. Then, they created a single draft version of the HSC scale in Spanish. Second, a third collaborative translator provided an unbiased opinion, and they compared all the translated versions. This process was completed when every item on measure was successfully synthesized. Third, two new bilingual translators in both languages translated the Spanish draft into the original language, creating a final English version. Fourth, the last two translators and another unbiased collaborator compared the two back-translated versions to the original measure to see if they were linguistically equal. Target culture was considered. Fifth, a Spanish research committee of four experts in sensory processing sensitivity issues aimed to ensure both the linguistic and cultural accuracy of the translated measure. Any discrepancies were resolved by consulting the whole process. Finally, cognitive interviews were conducted, which showed that all the items were easily understood. The interviewees did not have any difficulties with the response alternatives and their overall assessment regarding the instrument was positive. None of the interviewees said that they thought the inclusion of any other items was necessary.

### 2.3. Sample

The participants were selected through a non-probabilistic convenience sample of individuals from kindergarten and primary educative centers, which are representative of the Spanish context. The selection was carried out between December 2020 and February 2021. The following inclusion criteria were applied: (a) parents of children between 3 and 10 years old, and (b) parents of children schooled in kindergarten and primary educative centers. Parents were selected to complete the instruments and the following inclusion criteria were applied: (a) adults aged 18 years old or more; (b) having a child schooled in a kindergarten or primary educative center; and (c) adequate reading comprehension for the accomplishment of the evaluation protocol. Parents with sensory, physical or psychological deficits that make it difficult for the participant to understand and complete the evaluation instruments were excluded. The parents of children who present any neurodevelopmental disorder, autism or a sensory modulation disorder were excluded as well.

The sample was composed of 141 children (*n* = 141). The average age of the children was 6.75 years (*SD* ± 2.27). Out of the total number of children, 51.8% were boys (*n* = 73) and 48.2% were girls (*n* = 68). More than half of the sample was in primary education (62.4%), and 37.6% of the children were schooled at a kindergarten level.

### 2.4. Instruments

We used an ad hoc questionnaire that we created to collect sociodemographic and clinical data. The sociodemographic data for children considered in this study were: age, sex, and educational level (kindergarten or primary school).

The Highly Sensitive Child Scale (HSCS) [1], as indicated, is an instrument that assesses the environmental sensitivity of children, developed and tested for its psychometric properties by Pluess et al. [1]. It contains 12 items grouped in 3 subscales: (a) Ease of Excitation (EOE), (b) Aesthetic Sensitivity (AES) and (c) Low Sensory Threshold (LST). Each item was evaluated on a 7-point Likert-type scale, ranging from (1) ‘Strongly disagree’ to (7) ‘Strongly agree’ [32]. The internal consistency of the original HSC scale total score was *α* = 0.79 and the HSC subscales presented acceptable reliability scores with *α* = 0.71 for EOE, *α* = 0.73 for AES, and *α* = 0.66 for LST [1].

The Emotionality Activity and Sociality Survey (EAS) [33], which was validated in Spain [34], is an instrument that evaluates the temperament of children. It includes twenty items, which are divided into four groups: sociability, activity, emotionality, and shyness. Each item was evaluated on a 5-point Likert-type scale, ranging from (1) ‘Very uncharacteristic of the child’ to (5) ‘Very characteristic of the child’. The internal consistency estimated by the Spanish adaptation of the EAS was *α* = 0.51 for the total score, *α* = 0.31 for sociability, *α* = 0.62 for activity, *α* = 0.62 for emotionality, and *α* = 0.68 for shyness [34].

### 2.5. Procedure

First, to carry out the study, we contacted the principals of the education centers to inform them of main aim of the present research. After the school management teams were informed about the purpose of the study, meetings were held with parents of the students enrolled in kindergarten and primary education. During these meetings, parents were informed about the objectives of the study and that participation was voluntary. The parents who were interested in the study and those who met the inclusion criteria signed the informed consent. After the informed consent was signed by each parent, researchers gave them instructions on how to complete the questionnaires and resolved any doubts. Moreover, the instructions stated that only the mother or the father, the parent who spent more time with the child and profoundly understood the child’s behavior and personality, would be the one to complete the questionnaire [29]. Finally, 52.29% of the participants were mothers and 47.71% were fathers.

Although the present study was conducted in Spain, it was part of the European Project, so that it was approved by the Bioethics Committee of the University of Economics and Innovation in Lublin (16 December 2019) following the recommendations set out in the Ethical Principles for Medical Research involving human subjects [35]. To protect the strict confidentiality of the data, codes were assigned anonymously to identify the participants. All the participants were informed about the purpose of the study and the confidentiality of the data collected, and then signed their informed consent forms before participating.

### 2.6. Data Analysis

Confirmatory factor analyses (CFAs) were carried out in R (R Development Core Team, Vienna, Austria) [36,37,38] using the Maximum Likelihood Robust (MLR) estimation to deal with non-normality [39]. Following the original structure of the questionnaire, the fit of two models was compared: a one-factor model (HSC as a general factor) and a three-factor model (with three subscales as factors). The model fit was considered satisfactory if Yuan–Bentler χ^2^ was preferably non-significant (*p* > 0.05), the Comparative Fit Index (CFI) was 0.90 or above, the Root-Mean-Square Error of Approximation (RMSEA) was between 0.05 and 0.08, and the Standardized Root Mean Square Residual (SRMR) index was 0.08 or below [40,41,42].

We also examined the internal consistency of the total score and the subscales using Cronbach’s alpha (*α*) and McDonald’s omega (*ω*) in R [36]. Despite the fact that Cronbach’s alpha (*α*) is the most common coefficient to evaluate and estimate internal consistency, omega (*ω*) [43] is a factor analytic model-based coefficient of internal consistency that does not rely on tau-equivalence assumptions (i.e., unidimensionality, equal variances and covariances of the expected scores for the items) [44]. We considered Cronbach’s *α*’s of 0.60 or lower as low, between 0.60 and 0.70 as acceptable, and 0.70 or higher as good [45]. For *ω*, we regarded the values of 0.60 or lower as low, between 0.60 and 0.70 as acceptable, and 0.70 or higher as good [43].

Concerning the convergent validity, we analyzed bivariate correlations between the HSC total score and its subscales, and different dimensions of temperament in the Spanish sample using Jamovi (1.6 version) [46]. Correlations were considered as significant when *p* < 0.05.

Finally, we examined the differences between males and females, and between kindergarten and primary school education on environmental sensitivity. First, normality was checked with the Kolmogorov–Smirnov test and we decided to apply Student’s *t*-test [47,48]. Differences were considered statistically significant when *p* < 0.05. Moreover, the effect size was calculated to further understand the clinical relevance of these results. Cohen’s d criteria [48] were considered to represent the effect size, in which values of 0.49 or lower were regarded as small, between 0.50 and 0.79 as medium, and 0.80 or higher as large.

## 3. Results

### 3.1. Descriptive Analysis and Scale Performance

Descriptive statistics were calculated for each item of the HSC scale: ranges, means, standard deviations and percentiles. A 7-Likert scale response was used, and a floor effect was observed (6/1) for most items. The results are shown in Table 1.

### 3.2. Confirmatory Factorial Analysis

**Figure 1:**
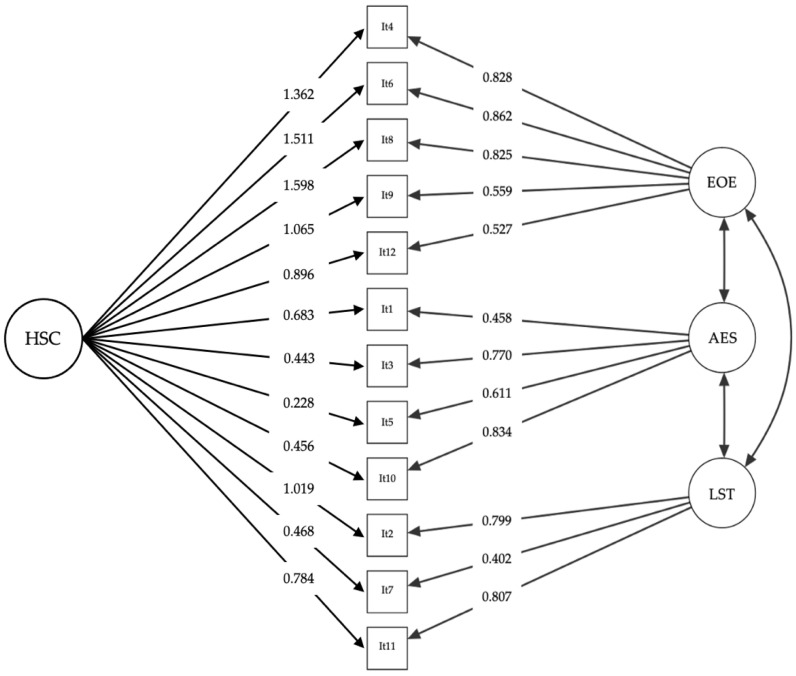
Factor loadings of the one-factor and the three-factor structures of the Spanish version of the HSC scale (*N* = 141). EOE = Ease of Excitation: It4, It6, It8, It9, and It12. LST = Low Sensory Threshold: It2, It7, and It11. AES = Aesthetic Sensitivity: It1, It3, It5, It and It10. HSC = General sensitivity factor: It1–It12.

First, a CFA was carried out to examine the bifactor structure found by Pluess et al. [1]. The results correspond to the bifactor model (i.e., general factor of sensitivity and the three dimensions), the one-factor model (i.e., HSC as a single factor) and three-factor model of the HSC scale in the sample of Spanish parents. The data show a satisfactory fit of the bifactor model, χ^2^(36) = 59.615, CFI = 0.97, TLI = 0.94, RMSEA = 0.07, SRMR = 0.04, 95% CI (0.04, 0.10) (Table 2). On the other hand, although the one-factor model did not show an adequate fit to the original one, the results reveal good fit indicators of the three-factor structure, χ^2^(51) = 99.517, CFI = 0.93, TLI = 0.91, RMSEA = 0.08, SRMR = 0.07, 95% CI (0.05, 0.11). The factorial loadings of the three-factor structure of most items exceed 0.45, with a range from 0.458 to 0.962, except for item 7, which shows a factorial loading of 0.402 (Figure 1). However, we decided not to remove any items due to the brevity of the scale and the contents of them, in order to maintain the original structure.

The instrument presented the following three dimensions: (a) Ease of Excitation (EOE; items 4, 6, 8, 9, 12), which indicates the potential to be easily overwhelmed by external and internal demands; Aesthetic Sensitivity (AES; items 1, 3, 5, 10), which refers to the aesthetic awareness, being deeply moved by arts and music; and Low Sensory Threshold (LST; items 2, 7, 11), which reflects unpleasant sensory arousal from external stimuli.

### 3.3. Reliability

Most of the corrected item-total correlations are above 0.30, except for items 5 (some music can make him/her really happy) and 7 (he/she does not like watching TV programs that have a lot of violence in them), which are below 0.28 (Table 3). The overall internal consistency of the HSCS was adequate (*α* = 0.839; *ω* = 0.845). Regarding the three dimensions, Ease of Excitation presents the highest reliability score (Table 4). Moreover, it was observed that the reliability of the full scale improved very slightly if items 5 and 7 were removed.

### 3.4. Convergent Valdity

Table 5 shows the correlations between the EAS and HSC scale that provided evidence of convergent validity of this parent-report version. The correlation found between the HSC scale and the emotionality dimension of the EAS was the highest, providing evidence of validity, according to the criteria proposed in the European model for the evaluation of the quality of the tests [49]. Low correlations were also found between the HSC scale and the sociability, activity and shyness dimensions of the EAS.

### 3.5. Differences in the Environmental Sensitivity between Female and Male Children and Educative Level in Children

In relation to the differences in the environmental sensitivity between males and females, the results show that statistically significant differences are found in the aesthetic sensitivity dimension, t_(138)_ = 2.102; *p* =0.037, reaching a higher score in females. However, the effect size of these differences is small (Table 6).

Concerning the differences between education levels on environmental sensitivity, primary education children presented higher scores for the EOE, AES and HSC total scale. In fact, the effect sizes are medium (Table 7).

## 4. Discussion

Previous research studies showed evidence in relation to the differences among children in their environmental sensitivity, with some being more sensitive to both negative and positive environmental contexts and internal stimuli [2,5,14,50]. Providing accurate assessment instruments can ease the detection of the level of SPS in children, in order to find out the characteristics of the personality trait and its health and social implications [11]. This study aimed to carry out a transcultural adaptation of the Highly Sensitive Child Scale and analyze its psychometric properties in a sample of Spanish children. The results reveal the good fit of the bifactor model proposed by Pluess et al. [1]. Moreover, this article revealed the satisfactory fit of the three-factor structure model and evidence of adequate internal consistency and convergent validity for the Spanish version of the HSC scale.

Following Pluess et al. [1] and Weyn et al. [17], the fit of the bifactor model, with a general sensitivity factor and a three-factor structure, was tested. The Spanish version of the HSC scale showed the adequate fit of the bifactor model. This bifactor structure model of the HSC scale suggests that both the general sensitivity score and the three subscale scores are essential to measure environmental sensitivity in Spanish children. These results are consistent with the findings of Pluess et al. [1]. Moreover, the three-factor structure obtained good fit indices in the Spanish sample through the CFA. Despite the fact that the only well-adjusted model was the one that presented a three-factor structure, different internal consistency indices of the Spanish version of the HSC scale indicated that the total score and its three dimensions had an acceptable-to-good reliability. These results are in line with the findings of these authors, both as a general sensitivity factor and as a three-factor structure [1,17] (Table 5).

Regarding the convergent validity, the distinction among the subscales was observed in the association between the Aesthetic Sensitivity and EAS dimensions, and the correlations between the other HSC scale dimensions and temperament dimensions. These associations indicated that high levels of SPS and its domains were positively associated with a higher rate of temperament and emotionality, assessed through the parent-report EAS scale. Additionally, greater AES was associated with higher sociability and lower shyness in the evaluated children. According to Belsky and Pluess [14], environmental sensitivity seems to be related to both approaching behaviors towards positive environments, and withdrawal behaviors towards negative ones. This finding appears to be in line with the idea that individuals scoring a high SPS can be more sensitive to both positive and negative environmental stimuli [14,51].

Additionally, based on these results, it seems that Aesthetic Sensitivity reflects another aspect of environmental sensitivity in the Spanish sample. AES may show a sensitivity towards positive experiences than Ease of Excitation and Low Sensory Threshold, confirming that these latter two domains are more similar [51].

Concerning the comprehension of environmental sensitivity, according to Chawla [52], albeit cultural interpretations change among people and places, it seems Spanish parents could have a similar understanding due to the fact that the structure of the HSC scale and its items are maintained with respect to the original one [1,17]. Additionally, a minor difference was found between males and females on the AES, presenting higher scores for females. This finding is also consistent with the findings of other authors, since it appears that environmental sensitivity tends to differ between sex groups; however, these differences are not significant [1,53]. Moreover, the age of children also seems to indicate a relationship with environmental sensitivity. Our results show that primary education children are more likely to present high levels of environmental sensitivity, especially in the EOE, AES and HSC total score. Indeed, Pluess et al. [1] also found positive correlations between age and environmental sensitivity.

This study also has its limitations. First, the sample was quite small, which could make the results difficult to generalize. Additionally, the moderate and low correlations which provide evidence of convergent validity with this sample must be considered when interpretating the results. These correlations should therefore be interpreted with caution, since EAS is not a sensory processing sensitivity measure. As a matter of fact, although hetero-informed questionnaires tend towards social desirability and provide some bias in the results, the 12-item HSC scale was adapted for use as a parent-rated measure of the children’s sensitivity [1]. Furthermore, the parent-report HSC measure demonstrated a power of prediction of externalizing problems in children [1]. However, more future research studies are needed to analyze the factorial invariance to find out the influence of gender and age, and if the measurement properties are independent of these variables. Moreover, invariance analysis should be conducted to test the interpretation of environmental sensitivity across gender and age groups [17]. In addition, the Spanish HSC scale could present difficulties to determine the level of sensitivity in children, since the assessment of sensory processing sensitivity could be made more objective with cognitive, genetic or physiological markers [7].

On the other hand, the present study is the first investigation of the Spanish parent-version of the HSC scale as a measure of environmental sensitivity in children. This paper also adds new evidence about the usefulness of the HSC scale for the assessment of sensory processing sensitivity in children. Increasingly more countries [7,17,22] are currently validating this instrument for different cultures, revealing adequate psychometric properties and factorial support. Indeed, the findings of this research are characterized by a sophisticated psychometric approach that provides a gold standard instrument for the assessment of environmental sensitivity. Therefore, the present study offers to the scientific community a transcultural adaptation of a highly reliable tool, which allows for the identification of the sensory processing sensitivity trait.

## 5. Conclusions

Overall, previous recent studies stated the need for the assessment of the level of SPS traits [7], due to the high prevalence rates of high sensitivity in the general population [11,54]. In fact, researchers pointed out the impact of different levels in SPS on school performance, health and quality of life in children [7,55,56,57]. Therefore, assessment instruments have a great variety of advantages because they can be administered quickly and easily, reducing time and human costs [58]. Additionally, self-reported child measures are essential, despite the power of prediction of behavioral problems, and given the parents’ difficulties to interpret the sensitivity of their children [59].

The present pilot cross-cultural validation of the HSC scale provides additional guarantees of methodological rigor. The HSC scale appears to be adequate for Spanish children’s sample application for the parent-report version. This measure with high reliability and validity for the assessment of this personality trait could benefit researchers, policy makers and practitioners, that is, to understand and promote positive development, quality of life and well-being better in highly sensitive children in the Spanish context.

## Figures and Tables

**Table 1:** Performance of the scale and related normative data.

Items of the Highly Sensitive Child Scale (HSCS)	R	M (*SD*)	P25	P75	Floor Effect (%)	Ceiling Effect (%)
Item 1. The child notices when small things have changed in his/her environment	1–7	5.08 (±1.62)	4	6	15.0	23.6
Item 2. Loud noises make him/her feel uncomfortable	1–7	4.25 (±1.85)	3	6	22.1	15.0
Item 3. The child loves nice smells	1–7	5.56 (±1.33)	5	7	21.4	0
Item 4. The child gets nervous when he/she has to do a lot in little time	1–7	4.31 (±1.74)	3	6	17.9	10.7
Item 5. Some music can make him/her really happy	1–7	5.71 (±1.28)	5	7	19.3	0
Item 6. The child is annoyed when people try to get him/her to do too many things at one	1–7	4.71 (±1.77)	4	6	23.6	17.9
Item 7. The child does not like watching TV programs that have a lot of violence in them	1–7	4.76 (±2.04)	3	6	18.6	22.1
Item 8. The child finds it unpleasant to have a lot going on at once	1–7	4.39 (±1.82)	3	6	19.3	13.6
Item 9. The child does not like it when things change in his/her life	1–7	4.17 (±1.74)	3	6	17.1	9.3
Item 10. The child loves nice tastes	1–7	5.78 (±1.39)	5	7	16.4	0
Item 11. The child does not like loud noises	1–7	4.66 (±1.78)	3	6	12.9	18.6
Item 12. When someone observes him/her, he/she gets nervous. This makes him/her perform worse than normal	1–7	4.10 (±1.63)	3	5	17.9	20.7

Note. p25 = 25th percentile; p75 = 75th percentile. Abbreviations: M, mean; *SD*, standard deviation; R, range.

**Table 2:** Confirmatory factor analysis (*N* = 141).

Model	CFI	TLI	SRMR	RMSEA (95% CI)
Bifactor model	0.97	0.94	0.04	0.07 (0.04–0.10)
One-factor model	0.62	0.54	0.13	0.19 (0.17–0.21)
Three-factor model	0.93	0.91	0.07	0.08 (0.05–0.11)

Note. CFI = comparative fit index; TLI = Tucker–Lewis index; RMSEA = root-mean-square error of approximation; SRMR = standardized root mean square residual.

**Table 3:** Psychometric characteristics of the Spanish version of the HSC scale.

Item	r_it_^c^	*α*-i	*ω*-i
**1**	0.492	0.828	0.834
**2**	0.624	0.817	0.826
**3**	0.447	0.832	0.837
**4**	0.585	0.821	0.826
**5**	0.258	0.842	0.849
**6**	0.649	0.815	0.821
**7**	0.274	0.849	0.849
**8**	0.711	0.810	0.816
**9**	0.518	0.826	0.832
**10**	0.433	0.832	0.838
**11**	0.558	0.823	0.832
**12**	0.487	0.828	0.835

Note. r_it_^c^ = correlation of item-total test; *α*-i = reliability if the item is dropped; *ω*-i = reliability if the item is dropped.

**Table 4:** Internal consistency.

	Cronbach’s *α*	McDonald’s *ω*
**HSC total scale**	0.839	0.845
**EOE**	0.862	0.867
**AES**	0.772	0.790
**LST**	0.725	0.765

Note. HSC = Highly Sensitive Child Scale; EOE = Ease of Excitation; LST = Low Sensory Threshold; AES = Aesthetic Sensitivity.

**Table 5:** Psychometric characteristics of the Spanish version of the HSC scale.

	1.	2.	3.	4.	5.	6.	7.	8.	9.
1. EOE	-								
2. AES	0.307 ***	-							
3. LST	0.426 ***	0.315 ***	-						
4. HSC total score	0.847 ***	0.655 ***	0.735 ***	-					
5. Sociability	−0.015	0.291 ***	0.082	0.127	-				
6. Activity	−0.060	0.214 *	−0.062	0.019	0.387 ***	-			
7. Emotionality	0.351 ***	0.259 **	0.274 **	0.399 **	0.036	0.154	-		
8. Shyness	0.208 *	−0.221 **	0.130	0.089	−0.420 ***	−0.314 ***	0.029	-	
9. EAS total score	0.285 ***	0.233 **	0.227 **	0.333 ***	0.330 ***	0.587 ***	0.679 ***	0.297 ***	-

Note. HSC = Highly Sensitive Child Scale; EOE = Ease of Excitation; LST = Low Sensory Threshold; AES = Aesthetic Sensitivity; EAS = Temperament Survey. * *p* < 0.05; ** *p* < 0.01; *** *p* < 0.001.

**Table 6:** Differences between males and females regarding the environmental sensitivity in children.

	M (*SD*)		*t*	*p*	d
	Males	Females			
**HSC total scale**	4.83 (0.97)	4.75 (1.05)	–0.432	0.666	–0.073
**EOE**	4.46 (1.33)	4.20 (1.46)	–1.089	0.278	–0.184
**AES**	5.35 (1.09)	5.73 (1.06)	2.102	0.037	0.356
**LST**	4.74 (1.35)	4.36 (1.68)	–1.472	0.143	–0.249

**Table 7:** Differences between kindergarten and primary level education regarding the environmental sensitivity in children.

	M (*SD*)		*t*	*p*	d
	Kindergarten	Primary Education			
**HSC total scale**	4.41 (1.12)	5.02 (0.86)	–3.634	<0.001	–0.633
**EOE**	3.78 (1.45)	4.68 (1.26)	–3.881	<0.001	–0.676
**AES**	5.19 (1.14)	5.74 (1.01)	–2.956	0.004	–0.515
**LST**	4.42 (1.57)	4.64 (1.49)	–0.848	0.398	–0.148

## Data Availability

The data are not publicly available due to reasons concerning privacy of the subjects and since it belongs to an ongoing project.
